# Analyzing Factors Influencing Patient Selection of a Surgeon for Elective Surgery in Saudi Arabia: A Questionnaire-Based Survey

**DOI:** 10.7759/cureus.32124

**Published:** 2022-12-02

**Authors:** Mohammed A Alosaimi, Abdulaziz S Alhamyani, Albaraa M Aljuaid, Amal A Aljuaid, Lama T Althobaiti, Fahad A Alosaimi, Tamer M Abdelrahman

**Affiliations:** 1 College of Medicine, Taif University, Taif, SAU; 2 Surgery, College of Medicine, Taif University, Taif, SAU; 3 Surgery, Benha Teaching Hospital, Benha, EGY

**Keywords:** saudi arabia, elective surgery, surgeon, selection, patient, influencing, factors

## Abstract

Background: Nowadays patients are encouraged to partake in healthcare decision making and patient preferences are given increasing weight. Patient’s choice is important to reduce waiting time and to encourage competition between providers, as most patients look for high-quality care while minimizing costs, according to different studies this may not be as simple as the attributes and factors that patients value when selecting a hospital or surgeon. Overall, Saudi Arabia has minimal research on how patients select surgeons or surgical facilities. Therefore, the goal of the current study was to evaluate the criteria Saudi population patients used to select their surgeons.

Objective: The objective is to determine the relative importance of the following aspects that patients may consider when selecting a surgeon: (a) physician-related considerations; (b) elements relating to healthcare service and access; (c) the perspectives of patients on various factors.

Methods: This observational cross-sectional study attempts to investigate the criteria that people in Saudi Arabia considered most important while selecting their surgeons. This study was conducted between August and November 2022. The questionnaire is also given in English along with Arabic.

Results: Six hundred nine completed the survey with a response rate of 91.6%. Concerning physician-related factors considered when choosing a surgeon attitudes of the physician were the factor most significantly contributed to physician selection rather than reputation or professional experience and physician social media: (84.7%) mentioned that paying attention to patient’s needs and opinions is important; sparing enough time for patients is important as reported by 83.9% of the participants; Personal care and hygiene is important for 83.4% of the participants; Communication skills were considered to be important as stated by 82.6% of the participants. Regarding healthcare services/access-related factors considered by patients in physician selection; it was found that patients considered hospital hygiene-cleanliness (91.3 %) as the most significant healthcare service/access-related factor in physician selection and then it comes Ease of obtaining an appointment (89.7%), reasonable scheduling and wait for time (87.7%) and cost of surgery (82.1%). Physician selection was deemed crucial to the success of the therapy by 87.7% of patients, while 88.3% of patients thought it was crucial to research the doctor before contacting her or him for the current admission.

Conclusion: Patients consider a wide range of variables when selecting their surgeons and the location of their procedure. Depending on sociodemographic, cultural, and other aspects, the conditions surrounding patients' decisions may vary. Overall, the selection of a surgeon by surgery patients appears to be more influenced by the doctor's attitudes than by his or her reputation, professional experience, or social media presence. In order to elicit preferences across a wider range of surgical subspecialties and patient demographics, more study is necessary.

## Introduction

In recent years, patients' roles have undergone a tremendous shift. Patients are encouraged to participate in making healthcare decisions and Patient preferences are being given more importance [1]. Reducing wait times and fostering provider competition were key factors in favoring patient choice, this selection method will encourage providers to compete for consumers by raising their quality and lowering their costs, as the majority of patients seek high-quality care while reducing expenditures [2]. However, based on numerous studies the qualities and criteria that patients value when choosing a hospital or surgeon may vary from population to population and from community to community, this may not be as straightforward as it first appears [3-5]. According to earlier research, patients should consider both patient-provider and healthcare center features when making their decision about a healthcare provider [2,6].

In general, patients consider a variety of criteria while selecting surgical services. The most coveted professional qualities of surgeons stood out as their reputation and competence. Additionally, patients frequently choose specialists based on their interpersonal skills [7,8]. With a wide range of healthcare options, choosing a practitioner is dependent more on recommendations from friends, family, and personal doctors than it is on newspaper rankings and social media ratings [8-10]. Another study's findings showed that the presence of a subspecialty certificate in surgery and the doctor's communications skills were the primary variables in patients' decisions on which doctors to choose [10]. The majority of participants thought that while the doctors' appearance was significant, compassion, politeness, and knowledge were more important [11]. Patients undergoing elective surgery understand how crucial it is to select a hospital, and they do so. Usually, patients have ample time to gather information prior to admission, and the three key considerations are personal hospital experience, proximity to their residences, and the hospital's level of expertise [12].

Schwartz reported that on the basis of mortality figures, some respondents asserted they would move hospitals for elective surgery. Since most respondents relied on their referring physician’s opinion to decide where to have surgery, surgical performance data ought to be accessible to referring physicians [13]. Although the majority of patients claimed to have chosen a hospital based on the institution's positive reputation (69.1%) and friendly hospital environment (63.3%) [14]. According to Aydin, the most important aspect of healthcare service/access that influenced doctor choice was hospital hygiene. [10]. Patients who preferred shorter waiting times before surgery were typically more ready to travel to a hospital outside of their immediate area [15]. Additionally, patients who attended screening services were prepared to go farther than their local hospital to receive a service with superior results [16]. Strong preferences for local treatment were, however, also seen in other research. Some patient groups were so unwilling to travel further that they would wait several months for surgery if it meant they could receive treatment close to home, in order to receive care locally, some patients were also willing to accept higher risks of morbidity and mortality [17]. But most patients preferred a nearby hospital to minimize the time and distance traveled [18]. 

Mavis reported that regardless of specialty, the gender of doctors was one of the least significant factors for the majority of women [19], unlike another study, which found that female patients favor female surgeons for breast surgeries or numerous treatments involving the breast [20]. However, the majority of patients questioned indicated they did not care about the possible surgeon's age, gender, color, or religion. Patients with preferences in these categories, however, frequently picked surgeons who shared their age, color, and religion [21]. According to Kurup, only 19% of the patients looked up a specific hospital online, compared to 32% who looked up a specific surgeon. However, specialists have found that online information on operations is often inconsistent and frequently of doubtful quality [22,23]. 

Overall, Saudi Arabia has minimal research on how patients select surgeons or surgical facilities. Therefore, the goal of the current study was to evaluate the criteria Saudi population patients used to select their surgeons.

## Materials and methods

Study design 

This observational cross-sectional study attempts to investigate the criteria that people in Saudi Arabia considered most important while selecting their surgeons. This study was conducted in 2022 between August and November. 

Study population and sampling methodology

This study includes a random sample of patients who came to be evaluated for all types of elective operations in different surgical clinics and that was in Taif hospitals, and the study population may be patients who underwent elective operations in clinics. The number of those who underwent surgical operations in Saudi Arabia reached (500,000) patients in the year 2021 according to the statistical report by the Ministry of Health, Saudi Arabia. The sample size is 600, the confidence level is 95%, and the margin of error is 5%. Data were collected by a randomized, self-administered questionnaire, which was sent to the respondents electronically via e-mail. Patients who did not agree to this study were excluded, and patients who either could not choose their surgeon or who were admitted to the hospital for emergency operations were also excluded. 

This questionnaire which consists of 42 questions was based on former studies and modification was done to be applied in our locality [10]. The study included the following elements in the questionnaire form: 1- The socio-demographic characteristics of the patients: including their age, level of education, gender, occupation, and economic situation. 2- Factors that contribute to the selection of surgeons by patients. A) Physician-related factors: the physician's reputation (recognition, advertisements, recommendations by other physicians/family members of friends/patients), attitudes (providing sufficient time for patients, communication skills, concern for patients' needs/opinions, kindness, sense of humor, personal care and hygiene) and Professional experience (age, years in practice, alma mater, an abroad experience, academic position, subspecialty certificate in surgery), and social media presence (personal website, sharing healthcare information, comments and ratings of users). Patients rated the importance of each of these factors in choosing a physician on a Likert scale (Not important, moderately important, and Important). B) Healthcare service/access-related factors are a contract with a health insurance company (doctor, hospital), simplicity of getting an appointment, reasonable scheduling and waiting time, cost of surgery, and hospital characteristics (recognition, environment, cleanliness/cleanliness, and location) elements. Patients rated the importance of each of these factors in choosing a physician on a Likert scale (Not important, moderately important, and Important). 3- Patients' view of factors contributing to a physician's choice of eight relevant criteria was assessed using a Likert scale to measure patient consent for each component (disagree, neutral, agree).

Data analysis

Statistical analysis was done using IBM SPSS Statistics for Windows, version 23.0 (IBM Corp., Armonk, NY). Continuous variables will be presented as mean ± standard deviation (SD) while categorical variables will be presented as numbers/percentages. Independent t-tests on both groups (e.g., males and females) and the ANOVA test were conducted to test the differences between groups larger than two groups (e.g., education level). P ≤ 0.05 was considered statistically significant.

Ethical considerations

The research protocol was approved by the Taif University research ethics committee (No. 43-767). The confidentiality of participants was preserved by not including names or identifying details in the proforma.

## Results

Socio-demographic data

Table 1 shows a total of 665 patients who met the inclusion criteria were approached to participate in this study; 609 approved and completed the survey in full, resulting in a response rate of 91.6%. In regard to age groups, Table 1 shows most of the participants 348 (57.1%) were within the age group of 18-30 years old and 372 (61.1%) were females. Regarding educational level, most of the participants 422 (69.3%) were with higher educational levels. Most of the participants 333 (54.7%) were students in terms of occupational status, 194 (31.9%) were employed, 51 (8.4%) were housewives and 31 (5.1%) were retired.

**Table 1:** Socio-demographic characteristics of the respondents (n=609)

Variable	Categories	N (%)
Age (years)	Less than 18	76 (12.5%)
	18 - 30	348 (57.1%)
	20 - 40	73 (12%)
	40 - 50	78 (12.8%)
	More than 50	34 (5.6%)
Gender	Male	237 (38.9%)
	Female	372 (61.1%)
Educational level	Primary education	3 (0.5%)
	Secondary education	148 (24.3%)
	Intermediate education	36 (5.9%)
	Higher education	422 (69.3%)
Occupational status	Student	333 (54.7%)
	Employed	194 (31.9%)
	Retired	31 (5.1%)
	Housewife	51 (8.4%)
Economic status (SAR)	Less than 5,000	366 (60.1%)
	5,000 – 10,000	115 (18.9%)
	10,000 – 20,000	106 (17.4%)
	More than 20,000	22 (3.6%)

Physician-related factors considered by patients in surgeon selection

Concerning physician-related factors considered when choosing a surgeon, Table 2 shows that attitudes of the physician were the factor most significantly contributed to physician selection rather than reputation or professional experience and physician social media: 84.7% mentioned that paying attention to patient’s needs and opinions is important; sparing enough time for patients is important as reported by 83.9% of the participants; personal care and hygiene is important for (83.4%) of the participants; communication skills were considered to be important as stated by 82.6% of the participants. Concerning the reputation of the physician: recommendation of a physician by his/her former patients was considered important by a higher percentage (56.8%) of patients in their provider selection, recommendation by another physician (31.9%), and recommendation by family members of friends (31.5%) rather than recognition or advertisements. The availability of a subspecialty certificate in surgery was deemed to be the most important factor (80%) influencing the choice of a physician with regard to professional experience. Then it comes in order of importance academic position (44.7%), years in practice (41.4%), and abroad experience (37.6%). In terms of the doctor's social media presence, more patients believed that user reviews and comments (66%) were very significant when choosing a doctor than the doctor's sharing of healthcare information (61.2%) or the doctor's presence on a personal website (43.2%).

**Table 2:** Physician-related factors that patients consider when choosing a surgeon

Factors	Not important	Moderately important	Important
	n (%)		
Reputation of the physician			
Recognition	204 (33.5)	229 (37.6)	176 (28.9)
Advertisements	20 (3.3)	451 (74.1)	138 (22.7)
Recommendation by another physician	218 (35.8)	197 (32.3)	194 (31.9)
Recommendation by family members of friends	36 (5.9)	381 (62.6)	192 (31.5)
Recommendation by his/her patients	146 (24)	117 (19.2)	346 (56.8)
Attitudes of the physician			
Sparing enough time for patients	7 (1.1)	91 (14.9)	511 (83.9)
Communicative skills	12 (2)	94 (15.4)	503 (82.6)
Paying attention to patient's needs/opinions	4 (0.7)	89 (14.6)	516 (84.7)
Kindliness	20 (3.3)	112 (18.4)	477 (78.3)
Good-humor	103 (16.9)	215 (35.3)	291 (47.8)
Personal care and hygiene	8 (1.3)	93 (15.3)	508 (83.4)
Professional experience of the physician			
Age	139 (22.8)	317 (52.1)	153 (25.1)
Years in practice	150 (24.6)	207 (34)	252 (41.4)
Alma mater	36 (5.9)	381 (62.6)	192 (31.5)
An abroad experience	176 (28.9)	204 (33.5)	229 (37.6)
Academic position	135 (22.2)	202 (33.2)	272 (44.7)
Subspecialty certificate in surgery	22 (3.6)	100 (16.4)	487 (80)
Social media presence			
Presence of personal website	209 (34.3)	137 (22.5)	263 (43.2)
Sharing healthcare information	51 (8.4)	185 (30.4)	373 (61.2)
Comments and ratings of users	43 (7.1)	164 (26.9)	402 (66)

Healthcare service/access-related factors considered by patients in physician selection

Regarding Table 3, the healthcare services/access-related factors considered by patients in physician selection; it was found that patients considered hospital hygiene-cleanliness (91.3%) as the most significant healthcare service/access-related factor in physician selection and then it comes to Ease of obtaining an appointment (89.7%), reasonable scheduling and wait for time (87.7%) and cost of surgery (82.1%). 

**Table 3:** Healthcare service/access-related factors considered by patients in physician selection

Factors	Not important	Moderately important	Important
	n (%)		
Contracted with the insurance company (Physician)	139 (22.8)	153 (25.1)	317 (52.1)
Contracted with the insurance company (Hospital)	117 (19.2)	146 (24)	346 (56.8)
Ease of obtaining an appointment	9 (1.5)	54 (8.9)	546 (89.7)
Reasonable scheduling and wait time	6 (1)	69 (11.3)	534 (87.7)
Cost of surgery	16 (2.6)	93 (15.3)	500 (82.1)
Hospital characteristics (Overall)	11 (1.8)	122 (20)	476 (78.2)
Hospital characteristics (Friendly atmosphere)	23 (3.8)	144 (23.6)	442 (72.6)
Hospital characteristics (Hygiene-cleanliness)	3 (0.5)	50 (8.2)	556 (91.3)
Hospital characteristics (Location)	52 (8.5)	151 (24.8)	406 (66.7)

Table 4 shows physician selection was deemed crucial to the success of the therapy by (87.7%) of patients, while (88.3%) of patients thought it was crucial to research the doctor before contacting her or him for the current admission. While the factors with which patients most frequently disagreed were the hospital's greater role in treatment outcomes than the doctor's (67.7%), the security of online sources for medical information (51.2%), and the value of watching online videos before surgery (47.5%).

**Table 4:** Patients' view on factors contributing to physician selection

Factors	Disagree	Neutral	Agree
	n (%)		
Physician selection is of utmost importance in the treatment outcome	8 (1.3)	67 (11)	534 (87.7)
As I performed during current admission, getting information about the physician is important	3 (0.5)	68 (11.2)	538 (88.3)
The hospital rather than the physician is important in the treatment outcome	412 (67.7)	43 (7.1)	154 (25.3)
There is difference between therapeutic approaches adopted by each physician	10 (1.6)	430 (70.6)	169 (27.8)
Getting information about treatment methods before contacting a physician is important	265 (43.5)	185 (30.4)	159 (26.1)
Online resources are safe source of healthcare information for patients	144 (23.6)	153 (25.1)	312 (51.2)
Watching online videos for the required surgical operation is useful	128 (21)	192 (31.5)	289 (47.5)

Association between socio-demographic data and the relative importance of the aspects that patients may consider when selecting a surgeon

Regarding Table 5, age was found to be significantly associated with physician-related factors, healthcare, and patient’s perspective factors (p-value ˂ 0.001, ˂ 0.001, and ˂ 0.001, respectively). With less than 18 years old group considered those previously mentioned factors more important than other groups. Gender and physician, healthcare-related factors, and patient’s perspective factors (p-value ˂ 0.001, 0.002, and 0.005, respectively) with female gender were found to be considering the previously mentioned factors as important more than men. Educational level was found to be significantly associated with physician-related, healthcare-related factors, and patient perspective factors (p-value ˂ 0.001, 0.003, and 0.001, respectively) with intermediate educational level more considering the previously mentioned factors as important as other educational levels. Occupational status was only found to be associated with healthcare related factors (p-value = 0.020) with housewives found to be more consider healthcare service-related factors as important more than others. Economic status was not found to be associated with a physician, health service, and patients' perspectives (p-value = 0.311, 0.145, and 0.190, respectively).

**Table 5:** Association between socio-demographic data and factors considered by patients in surgeon selection.

Variable	Categories	Physician related factors		Healthcare service / access-related factors		Patients' perspectives on many factors	
Age (years)	Less than 18	2.73 (0.309)	< 0.001	2.85 (0.261)	< 0.001	2.77 (0.351)	< 0.001
	18 - 30	2.49 (0.346)		2.67 (0.343)		2.52 (0.387)	
	20 - 40	2.59 (0.297)		2.76 (0.314)		2.60 (0.388)	
	40 - 50	2.51 (0.304)		2.57 (0.342)		2.40 (0.401)	
	More than 50	2.49 (0.322)		2.59 (0.348)		2.41 (0.307)	
Gender	Male	2.45 (0.348)	< 0.001	2.63 (0.367)	0.002	2.48 (0.407)	0.005
	Female	2.59 (0.321)		2.72 (0.315)		2.57 (0.381)	
Educational level	Primary education	2.50 (0.477)	< 0.001	2.56 (0.444)	0.003	2.48 (0.541)	< 0.001
	Secondary education	2.52 (0.371)		2.67 (0.378)		2.57 (0.410)	
	Intermediate education	2.78 (0.294)		2.89 (0.247)		2.79 (0.304)	
	Higher education	2.52 (0.322)		2.67 (0.326)		2.50 (0.386)	
Occupational status	Student	2.53 (0.351)	0.986	2.71 (0.331)	0.020	2.57 (0.393)	0.104
	Employed	2.53 (0.321)		2.64 (0.341)		2.51 (0.381)	
	Retired	2.51 (0.393)		2.58 (0.455)		2.42 (0.451)	
	Housewife	2.53 (0.292)		2.74 (0.274)		2.49 (0.391)	
Economic status (SAR)	Less than 5,000	2.52 (0.351)	0.311	2.69 (0.345)	0.145	2.55 (0.392)	0.190
	5,000 – 10,000	2.58 (0.313)		2.72 (0.303)		2.55 (0.391)	
	10,000 – 20,000	2.53 (0.307)		2.62 (0.341)		2.46 (0.399)	
	More than 20,000	2.56 (0.381)		2.69 (0.384)		2.57 (0.382)	

## Discussion

Assessing factors influencing a patient’s selection of a surgeon for elective surgery is important as patient comfort nowadays is counted as one of the indicators of healthcare service quality and psychological status was found to be having effects on response to treatment as mentioned in many studies [24,25]. It is obvious that patients' decisions to choose a surgeon or institution for surgical care are complex and heterogeneous. There is not just one set of criteria that patients must consider while making decisions. Overall, it seems that clinical considerations had the most impact on patients. Patients prefer to be treated by surgeons who have solid reputations and possess both good interpersonal and technical skills. Patients also favored medical facilities with outstanding reputations and levels of care. With the exception of travel distance and waiting periods, nonclinical factors did not have a significant impact on patient choice. Patients employed a variety of information sources, and they seemed to value different sources differently depending on the situation [8].

In the present study, we found that the attitudes of the physician were the factor most significantly considered when choosing a surgeon rather than reputation or professional experience, and surgeons' social media were paying attention to patient’s needs and opinions, sparing enough time for patients and communication skills considered highly significant by the majority of the participants. These results are consistent with recent data from communities of surgery patients, which emphasize that despite the surgeon's propensity to concentrate largely on surgical quality, technical competence, and risk-adjusted outcome indicators, patients actually place great significance on the relationship between the surgeon and patient [3,10,26,27]. This seems to point up the significance of surgeons regaining awareness of the patient's priorities associated with this fundamental component of patient care.

The reputation of the physician had an important role for the participants in our study and this was consistent with the study conducted by Gao et al. in which physician reputation was found to be of significant importance for the participants [28]. The recommendation of a physician by his/her former patients was considered the most important in our study while Harris reported in his that the most important is the recommendation by another physician [29]. Recommendation of a physician by his/her former patients, by another physician, and by family members or friends was considered more important by patients rather than recognition or advertisements. This appears to support the idea that a patient's decision about a provider is largely focused on their personal experiences or the recommendation of a doctor, family, or friend rather than on comparative consumer information.

In our study, the availability of a subspecialty certificate in surgery was deemed to be the most important factor influencing the choice of surgeons with regard to professional experience which is consistent with the past studies that indicated board certification and specialized training as the factors with the highest patient ratings in surgeon selection [26,30]. We also found that one of the factors affecting the patient's choice of surgeon is the academic position, years in practice, and abroad experience so we must take it into consideration. The age of the physician is important for only 25.1% of the participants this was in contrast to the findings reported in the study carried out by Manning et al. in which age was found to be important for more than half of the respondents [31]. In terms of the doctor's social media presence, more patients in our study believed that user reviews and comments (66%) were very significant when choosing a doctor than the doctor's sharing of healthcare information (61.2%) or the doctor's presence on a personal website (43.2%). This was found to be in contrast with the study conducted by Coulter et al. in which media and advertisements were not important for most of the participants [32].

Hospital hygiene and cleanliness were judged to be the most important healthcare service/access-related factors in patient selection (91.3%), according to research on the criteria patients examined when choosing a doctor. Similar to our results, hospital cleanliness was also reported to be the most important factor in hospital selection by Aydin and Gokcen in their study [10]. Then, it comes the ease of obtaining an appointment is considered to be important by 89.7% of the participants and the reasonable scheduling and wait time (87.7%), this was consistent with the findings of the study conducted by Burger et al. in which appointment availability as one of the most important determinant factors for physician selection [33]. Regarding being contracted with the insurance company for physicians and contracted with the insurance company for the hospital is important only by nearly half of our participants; similar findings were found in the Fareed's study which reported that insurance is one of the most important factors in physician selection [34]. The outcome of treatment is greatly affected by physician selection as agreed on by the vast majority (87.7%) of the participant's similar findings were reported in the congruent study carried out by Bozic et al. in which mentioned physician concentration on the outcome as it is considered as one of the most significant factors in physician selection [3].

Prior to contacting the majority of our patients' existing surgeons, it is critical to identify them and learn more about them. The selection process for our patients appears to be mostly driven by the doctor's demeanor, which is more likely to be attained from personal experience or referrals from previous patients, family members, or friends. Educational level was found to be significantly associated with physician-related, healthcare-related factors, and patient perspective factors, with intermediate educational level more considering the previously mentioned factors as important as other educational levels. Age was found to be significantly associated with physician-related factors, healthcare, and patient’s perspective, with the less than 18 years old group considering those previously mentioned factors more important than other groups. Gender and physician, healthcare-related factors, and patient’s perspective factors with female gender were found to be considering the previously mentioned factors as important more than men and this was found to be contradictory to the findings reported in the study conducted by Charles et al. in which age and gender were not found to be significantly associated with physician selection by most of the participants [35].

The majority of our patients researched their current surgeon online or through referrals from other patients, family members, or friends of other doctors before making contact with him or her. Our findings thus indicate that, at least based on their priorities, our patients are likely to lack impartial sources and comparative public reports for information that would be most helpful to them in selecting a surgeon. Given the alleged discrepancy between patients' desire for information on the quality of care and its incorporation into the decision-making process for choosing a healthcare provider, this is debatable.

## Conclusions

Patients consider a wide range of variables while selecting their surgeons and the location of their procedure. Depending on sociodemographic, cultural, and other aspects, the conditions surrounding patients' decisions may vary. Our research found that most patients believed that choosing the right doctor would have the biggest impact on how well their treatment went and that most of them had knowledge of their current surgeon before making contact with him or her, mostly thanks to personal experience or recommendations from previous clients. Overall, the selection of a surgeon by surgery patients appears to be more influenced by the doctor's attitudes than by his or her reputation, professional experience, or social media presence. In order to elicit preferences across a wider range of surgical subspecialties and patient demographics, more study is necessary, and providers will be able to help patients find their preferred caregiver with the aid of more information on how patients select surgeons or hospitals.
